# Prostatic artery embolization versus conventional TUR-P in the treatment of benign prostatic hyperplasia: protocol for a prospective randomized non-inferiority trial

**DOI:** 10.1186/1471-2490-14-94

**Published:** 2014-11-25

**Authors:** Dominik Abt, Livio Mordasini, Lukas Hechelhammer, Thomas M Kessler, Hans-Peter Schmid, Daniel S Engeler

**Affiliations:** Department of Urology, Cantonal Hospital St. Gallen, Rorschacherstrasse 95, St. Gallen, 9007 Switzerland; Department of Radiology and Nuclear Medicine, Cantonal Hospital St. Gallen, Rorschacherstrasse 95, St. Gallen, 9007 Switzerland; Neuro-Urology, Spinal Cord Injury Centre & Research, University of Zürich, Balgrist University Hospital, Forchstrasse 340, Zürich, 8008 Switzerland

**Keywords:** Prostate, Benign prostatic hyperplasia, Transurethral resection of the prostate, Embolization, Prostatic artery embolization, Comparative clinical trial

## Abstract

**Background:**

Benign prostatic hyperplasia (BPH) is a prevalent entity in elderly men and transurethral resection of the prostate (TURP) still represents the gold standard of surgical treatment despite its considerable perioperative morbidity. Recently, prostatic artery embolization (PAE) was described as a novel effective and less invasive treatment alternative. Despite promising first results, PAE still has to be considered experimental due to a lack of good quality studies. Prospective randomized controlled trials comparing PAE with TUR-P are highly warranted.

**Methods/design:**

This is a single-centre, prospective, randomized, non-inferiority trial comparing treatment effects and adverse events of PAE and TURP in a tertiary referral centre. One hundred patients who are electable for both treatment options are randomized to either PAE or TURP. Changes of the International Prostate Symptom Score (IPSS) after 3 months are defined as primary endpoint. Changes in bladder diaries, laboratory analyses, urodynamic investigations and standardised questionnaires are assessed as secondary outcome measures. In addition contrast-enhanced magnetic resonance imaging of the pelvis before and after the interventions will provide crucial information regarding morphological changes and vascularisation of the prostate. Adverse events will be assessed on every follow-up visit in both treatment arms according to the National Cancer Institute Common Terminology Criteria for Adverse events and the Clavien classification.

**Discussion:**

The aim of this study is to assess whether PAE represents a valid treatment alternative to TURP in patients suffering from BPH in terms of efficacy and safety.

**Trial registration:**

ClinicalTrials.gov NCT02054013.

## Background

Benign prostatic hyperplasia (BPH) is a prevalent entity, affecting over 50% of men older than 60 years [1]. The clinical picture of the disease includes lower urinary tract symptoms such as interrupted and weak urinary stream, nocturia, urgency and leaking and even sexual dysfunction in some individuals [2]. Medical therapy is usually the first-line treatment [3]. However, the efficacy of drugs like alpha-blockers is limited, and as disease progresses more invasive treatment options have to be taken into consideration.

In cases with moderate to severe lower urinary tract symptoms transurethral resection of the prostate (TURP) is still the standard treatment. TURP, however, is limited to prostates smaller than 60-80 ml and the procedure is associated with a substantial complication rate. The cumulative short-term morbidity rate is around 11% and the necessity for surgical revision is as high as 6%. Bleeding requiring transfusions and transurethral resection syndrome represent potentially serious threats to elderly and frail patients [4]. Prostatic artery embolization (PAE) has been suggested as a minimal invasive alternative procedure, which can be performed in an outpatient setting with rapid recovery and low morbidity [5, 6].

PAE was first described in 1979 by Lang et al. [7] as a treatment option in intractable prostatogenic haemorrhage and emerged as a safe and effective treatment thanks to technical refinements throughout the last decades. Using PAE in this purpose, DeMeritt et al. were the first to report on relief of BPH-related bladder outlet obstruction after transarterial polyvinyl alcohol prostate embolization in 2000 [8].

First intentional treatment of BPH by PAE was published in 2008 [9]. Subsequently, promising short- and medium-term results could be shown for patients with symptomatic BPH, refractory to medical treatment: A significant improvement in the International Prostate Symptom Score (IPSS) and maximum urinary flow rate, as well as a reduction of prostate volume and post-void residual urine were reported in several studies [10–13]. Methods and technique to perform PAE are well established and have been described in several publications [14, 15]. PAE was shown to be a safe procedure with low morbidity in carefully selected patients [16, 17].

However, data concerning PAE has been criticized for different reasons: There is just a small number of studies published by only three research groups with an unclear overlap of the patients that were described so far. Moreover, quality of the studies available was referred to be poor due to study type (cohort), unclear patient selections and dropouts as well as statistical limitations and missing long-term results [17].

Currently, only a single trial was published comparing TURP and PAE: Gao et al. report on promising results of PAE with a post-interventional course outshining the data published so far [18]. This study, however, was devoted little attention most likely due to ambiguities regarding patient selection and good clinical practice issues. TURP still remains clearly the gold standard in surgical treatment of BPH and a prospective randomized trial according to good clinical practice (GCP) comparing PAE and TURP is mandatory, to assess efficacy and safety of PAE in the treatment of BPH.

## Methods and design

### Study design and location

This is a prospective, randomized, non-inferiority trial conducted at the urological and radiological departments of Cantonal Hospital St. Gallen, St. Gallen, Switzerland.

### Study population and recruitment

Recruitment of the study participants is performed at the urological outpatient clinic of Cantonal Hospital St. Gallen by the principle investigator (PI). The PI will check for inclusion and exclusion criteria (Table 1) by reviewing the patient’s medical record and by patient-doctor conversation. Study participants are thoroughly informed about the study by the study physician. Possible questions are answered by the PI. If the patient feels well informed and confident to participate in the trial, informed consent can be given within the consultation. If the patient needs further time for consideration, an additional appointment in the outpatient clinic will be arranged within the next 2–3 weeks. Anyway, the patient has got at least 2–3 weeks till admission to the hospital to get clear on study participation and is able to ask further questions at the day of admission.

**Table 1:** **Inclusion and exclusion criteria**

Inclusion criteria	Exclusion criteria
• Men older than 40	• Severe atherosclerosis
• Patient must be a candidate for TURP	• Severe tortuosity in the aortic bifurcation or internal iliac arteries
• Refractory to medical therapy or patient is not willing to consider (further) medical treatment	• Acontractile detrusor
• Patient has a prostate size of at least 25 ml and not more than 80 ml, measured by ultrasound	• Neurogenic lower urinary tract dysfunction
• IPSS ≥8	• Urethral stenosis
• QoL ≥3	• Bladder diverticulum
• Qmax < 12 and/or urinary retention	• Bladder stone with surgical indication
• Written informed consent	• Allergy to intravenous contrast media
	• Contraindication for MRI imaging
	• Preinterventionally proven adenocarcinoma of the prostate
	• Renal failure (GFR < 60 ml/min)

### Study randomisation

Randomisation will be performed using SecuTrial (InterActive Systems GmbH, Berlin, Germany) stratifying on age (<70, ≥70 years) and prostate volume (<50 ml, ≥50 ml).

### Study procedures

**Figure 1:**
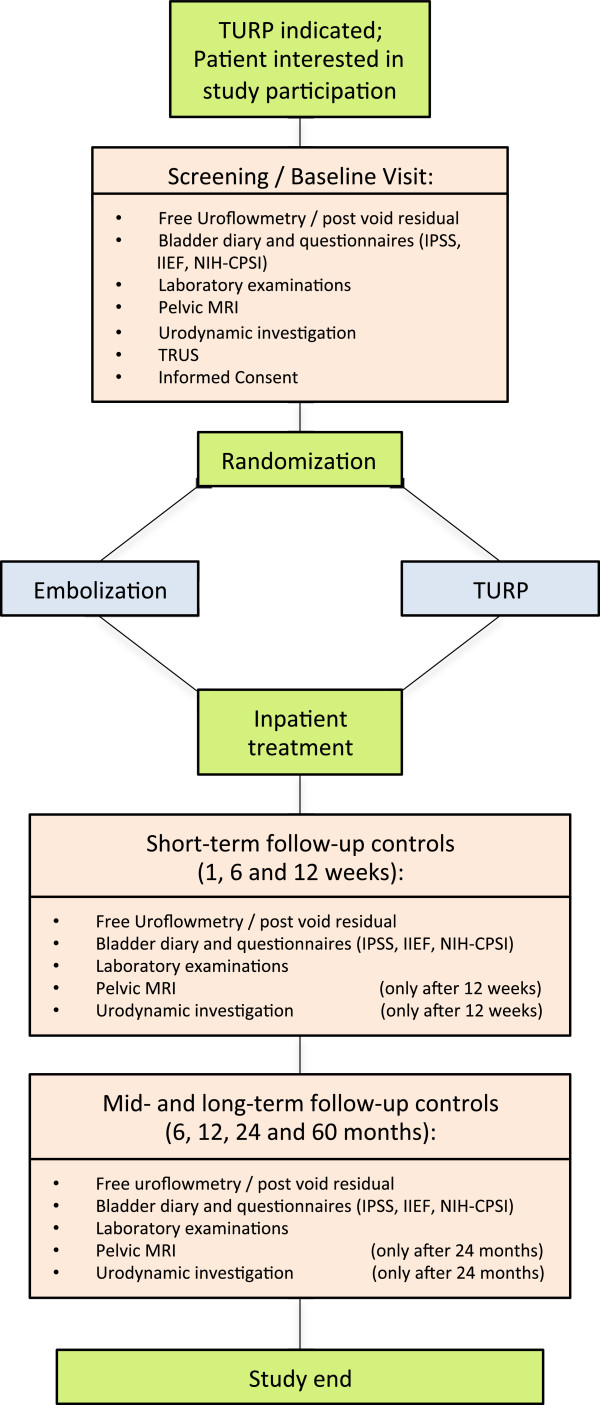
**Timetable and characteristics of study visits.**

After baseline visit participants are randomized to TURP or embolisation (Figure 1). Both interventions are performed in an inpatient setting. All subjects receive perioperative antibiotic prophylaxis started one day before procedure and continued for one day after catheter removal (Ciprofloxacin 500 mg twice daily, except infection with different resistance profile was proved before). Moreover, anti-inflammatory (Diclofenac 75 mg twice daily) and acid-suppressing medication (Pantoprazole 40 mg once daily) is administered for 1 week starting at the day of intervention. Prostatic medication is abandoned at the day of TURP and 2 weeks after PAE (due to supposed slower efficacy).

### TURP

For monopolar transurethral resection, a 24 F Storz resectoscope with a cutting power of 150 W and a coagulation power of 60 W is used. A standard tungsten wire loop (Karl Storz Endoskope; Anklin AG, Binnigen, Switzerland) and electrolyte-free mannitol-sorbitol solution (Purisole, Fresenius Kabi AG, Bad Homburg, Germany) will be used for TURP. Surgery will be performed under spinal or general anaesthesia according to patient’s and anaesthetist’s preferences by one of the physicians involved into the study (LM, DA, HPS, DSE). A 20 F three-way catheter is inserted for irrigation after resection and left for at least two days depending on bleeding tendency.

### PAE

A 16 F transurethral catheter is inserted prior to intervention for better radiological orientation. After local anaesthesia, a unilateral femoral sheath is placed (normally the right common femoral artery) and the patient will have a selective internal iliac arteriogram of the anterior division of both internal iliac arteries by a 5 F catheter to identify the prostatic arterial supply. In special anatomical variants, arteriograms of the external iliac and their branches will be performed. The prostatic vessels, which can derive from every branch of the anterior division, will be selectively catheterized with a 2–3 F micro catheter and subsequent embolization will be performed with 250-400 μm sized Embozene Microspheres (Celonova, San Antonio, TX). The embolization endpoint will be absence of perfusion of the prostate on post embolization angiography and stasis of flow in the prostate arteries. This procedure is performed on both sides whenever possible. Embolization will only be performed by a single interventional radiologist (LH). Transurethral catheter is removed on the first morning after intervention.

### Study outcome measures

#### Characteristics and timing of visits

Regular follow-up controls starting one week after intervention and continued up to 5 years will be performed, assessing the parameters described in Figure 1.

### Primary and secondary endpoints

Change of International Prostate Symptom Score (IPSS) 3 months after intervention was defined as primary endpoint. Secondary study endpoints are shown in Table 2.

**Table 2:** **Primary and secondary endpoints**

**Primary endpoint**	• Changes in the IPSS 12 weeks after intervention
**Secondary endpoints** (see Figure 1 for time points)	
	• Changes in free uroflowmetry and post-void residual
	• Changes in bladder diary
	• Changes in urodynamic investigation
	• Changes in IPSS, CPSI and IIEF
	• Changes of haemoglobin and serum PSA
	• Duration of post procedure catheterisation and hospitalisation
	• Procedure time and radiation parameters
	• Changes of prostate volume, measure of devascularized/resected tissue using MRI
	• Comparison of prostate size, measured preoperatively by TRUS and MRI at baseline

### Statistics, study sample size and power calculation

For the primary endpoint, changes in IPSS at 12 weeks will be compared using a one-sided t-test with significance level 0.025 (equivalent to using the boundaries from a 95% confidence interval (CI)). As long as the t-test is not significant (that is, the 95% CI is entirely above -3), PAE will be considered non-inferior to TUR-P. A second analysis of the primary endpoint will be adjusted for IPSS at baseline (using linear regression), and therefore an adjusted confidence interval will also be reported.

Mean differences and corresponding 95% CI will be reported for all secondary endpoints, as well as the p-value from a one-sided t-test. Where an endpoint is clearly not normally distributed, results from a one-sided Mann–Whitney U-test will be substituted. Changes over time will be compared pairwise in the same fashion.

In a study performed at our own institute [19], the standard deviation for IPSS was 4.6. A one-sided t-test with one-sided significance level 0.025 will have 80% power to reject the null hypothesis that the two treatments are not equivalent (that is, the difference in means is -3 or further from zero in the same direction), assuming the expected difference is 0 and the common standard deviation is 4.6, when the sample size is 38 patients in each group. Assuming a dropout rate of 20%, we aim to recruit 100 patients total.

### Regulatory issues

#### Ethical approval

Study was approved by the local ethics committees (EKSG 14/004) and is performed in consideration of the World Medical Association Declaration of Helsinki [20], the guidelines for GCP [21], and the guidelines of the Swiss Academy of Medical Sciences [22]. Handling of all personal data will strictly comply with the federal law of data protection in Switzerland [23].

### Quality control, quality assurance and confidentiality

An expert of Clinical Trials Unit (CTU) St. Gallen conducts data monitoring according to GCP. Trial-related monitoring, audits and regulatory inspections from the ethics committee (EKSG) will be permitted by the principal investigator, providing direct access to source documents. Data collection is performed using electronic case report forms (SecuTrial) programmed by CTU St. Gallen. The respect of the professional secrecy is guaranteed. Insight into the data collected in this trial will only be provided to the involved investigators, the members of the ethics committee experts responsible for the monitoring.

### Missing data

Patients will be included in the primary analysis of the primary endpoint, provided that baseline and 12 week IPSS measurements are available. For all other analyses, all collected data will be analyzed.

### Safety

All adverse events (AE) and serious adverse events (SAE) which might be related to the study procedures are collected, fully investigated and documented during the entire study period. Assessment of severity of all AEs will be performed according to National Cancer Institute Common Terminology Criteria for Adverse Events v4.0 (CTCAE) and according to the Clavien classification [24]. All SAEs related to study intervention, are reported to the local Ethics Committee. All AEs and SAEs will be followed as long as medically indicated.

## Discussion

The aim of this study is to assess whether PAE is a valuable treatment option compared to TURP in patients with BPH, assessing both, short- and long-term treatment effects as well as complications. Using a prospective, randomized, non-inferiority trial design with clearly defined endpoints, as well as inclusion and exclusion criteria and performed according to well-defined quality standards, data will help to estimate treatment efficiency of PAE better. Moreover, potential advantages as well as problems of this emerging intervention can be analysed. The study might also help to define patients that are particularly suitable for PAE and patients that should be treated alternatively. In addition, magnetic resonance imaging performed at different time intervals to intervention might help to get a better understanding of the underlying mechanisms.

### Trial status

The trial is in the recruiting phase at the time of manuscript submission.

## Abbreviations

BPH: Benign prostate hyperplasia
CI: Confidence interval
GCP: Good clinical practice
IIEF: International index of erectile function
IPSS: International prostate symptom score
MRI: Magnetic resonance imaging
NIH-CPSI: National institute of health – chronic prostatitis symptoms score
PAE: Prostatic artery embolization
PI: Principle investigator
PSA: Prostate specific antigen
TURP: Transurethral resection of the prostate
TRUS: Transrectal ultrasound.
